# Floorball goaltending movements during a game: A quantitative observational study

**DOI:** 10.1186/s13102-025-01202-6

**Published:** 2025-06-09

**Authors:** Eva Tengman, Alexandra Pettersson, Linnea Jönsson, Taru Tervo

**Affiliations:** 1 Department of Community Medicine and Rehabilitation, Section for Physiotherapy, Umeå University, Umeå, SE-90187 Sweden; 2 Umeå School of Sport Sciences, Umeå University, Umeå, Sweden; 3 Floorball Research and Development Centre, Umeå School of Sport Sciences, Umeå University, Umeå, Sweden

**Keywords:** Goalkeepers, Sport specific movements, Video analysis

## Abstract

**Background:**

More knowledge about floorball goalkeepers’ game movements and biomechanical demands is warranted. Therefore, the aim of this study was to observe type and frequency of female floorball goalkeepers’ movements during a game.

**Methods:**

Twelve female goalkeepers (mean age 22 years) were included. An observational study design using video recording was used to explore goalkeepers’ movements during a game. Three different positions were reported in minutes, percent of playing time, and the number of times the goalkeepers performed different movements.

**Results:**

The results revealed that of total playing time, goalkeepers were playing on their knees 31% (± 10%) of the time, in quadruped position 1.3% (± 1.9%) and 67% (± 11%) they played standing. Five movements were performed more frequently; short side movements, pull, stand up, small rotation with legs, and longer ball covers at the goalpost. A large variation in movement was seen regarding how many and what type of movements each goalkeeper performed during the game.

**Conclusion:**

Floorball goalkeepers perform a high number of movements and spend a substantial amount of the match time on their knees. There is no existing research on how these game aspects may impact load as well as physical needs, emphasizing the need for further research. Knowledge from the study lays the groundwork for further studies and may be used when developing future preventive training programme and rehabilitation.

## Background

Floorball is an indoor team sport which is played in over 80 countries worldwide, according to the International Floorball Federation [1]. It is a fast-paced game consisting of acceleration, sudden stops, and change of direction [2]. Data exists on floorball-related injuries showing that injury incidence both at junior and adult levels are high [3–6] and that females experience higher injury incidence rates than males [7]. Additionally, there is some studies on how to prevent acute injuries, to some extent, through injury preventive programs [8, 9]. However, when it comes to floorball goalkeepers, there is a lack of knowledge of biomechanical factors, physical demands, and separate injury incidence.

In floorball and other team sports, goalkeepers play an important role. Goalkeepers’ ability to defend the goal is one of the key aspects of a team`s overall performance. To perform well, the goalkeeper must have technical skills like speed, fast reactions, good hand–eye coordination, and rapid decision-making. Unique to floorball goalkeepers is that most of the movements made while defending the goal, are done on one’s knees. Several floorball goalkeepers choose to stand up while the match is played on opponent’s game plan, but as soon as a game changes direction, the goalkeeper takes on a more active role and changes position to their knees. The physical requirements for goalkeepers differ greatly from requirements for field players, as has described for other team sports like handball [10], ice hockey [11, 12], and soccer [13]. Therefore, it is important to explore floorball goalkeepers’ practice of the sport as well as their injuries separately from field players.

To the best of our knowledge, there are no previous studies on floorball goalkeepers’ movements during game. Studies observing floorball goalkeepers’ movements are important for a better understanding different movements and loads, as well as physical needs. With this knowledge it is possible to design and modify suitable training programmes which consider the specific physical needs of floorball goalkeepers.

Hence, the aim of this study was to observe type and frequency of female floorball goalkeepers’ movements during a game. We hypothesise that most of the movements made when defending the goal are done on the knees, involve a great number of short lateral movements, and show a large individual variation among goalkeepers.

## Methods

### Study design

A observational study design was used to explore female floorball goalkeepers’ type and frequency of movements during games. The study design allowed us to observe goalkeepers in their natural setting during games.

### Participants

Twelve female goalkeepers competing in high level floorball in Sweden were video recorded and observed during a game. Inclusion criteria were goalkeepers playing in division 1 North, where all ten teams were contacted by email for recruitment and all teams agreed to participate. Data collection was performed during the 2020 season. The floorball goalkeepers were aged between 16 and 32 years (mean 22 years SD 5 years), they had been practicing floorball for an average of 12 years (SD 4 years), and goalkeeping specifically for an average of 10 years (SD 4 years). Information regarding the study was first sent to the coaches by email which was then forwarded to the goalkeepers. This study followed the principles of the Declaration of Helsinki [14] and complied with the guidelines of the Swedish Ethical Review Authority. Since games are filmed, all players in the league have sign a consent for that. Further, before recording the games for the aim of this study, all goalkeepers received both oral and written information and participants gave their written informed consent. Age, and number of years as a goalkeeper were collected, and after inclusion participants were pseudonymised. No health-related information, or sensitive personal data was processed or collected from any participant. Ethics approval was deemed unnecessary according to national regulations, since no health-related data were collected, or no physical/psychological interventions were involved.

### Data collection

Games were recorded during a regular league match with an audience. Two video cameras were used during data collection; one camera recording each goalkeeper during the game. The cameras were placed on spectator stands and recorded the goalkeepers obliquely from the front while zooming in on both the goalkeeper and the goal area.

### Video analysis

**Fig. 1:**
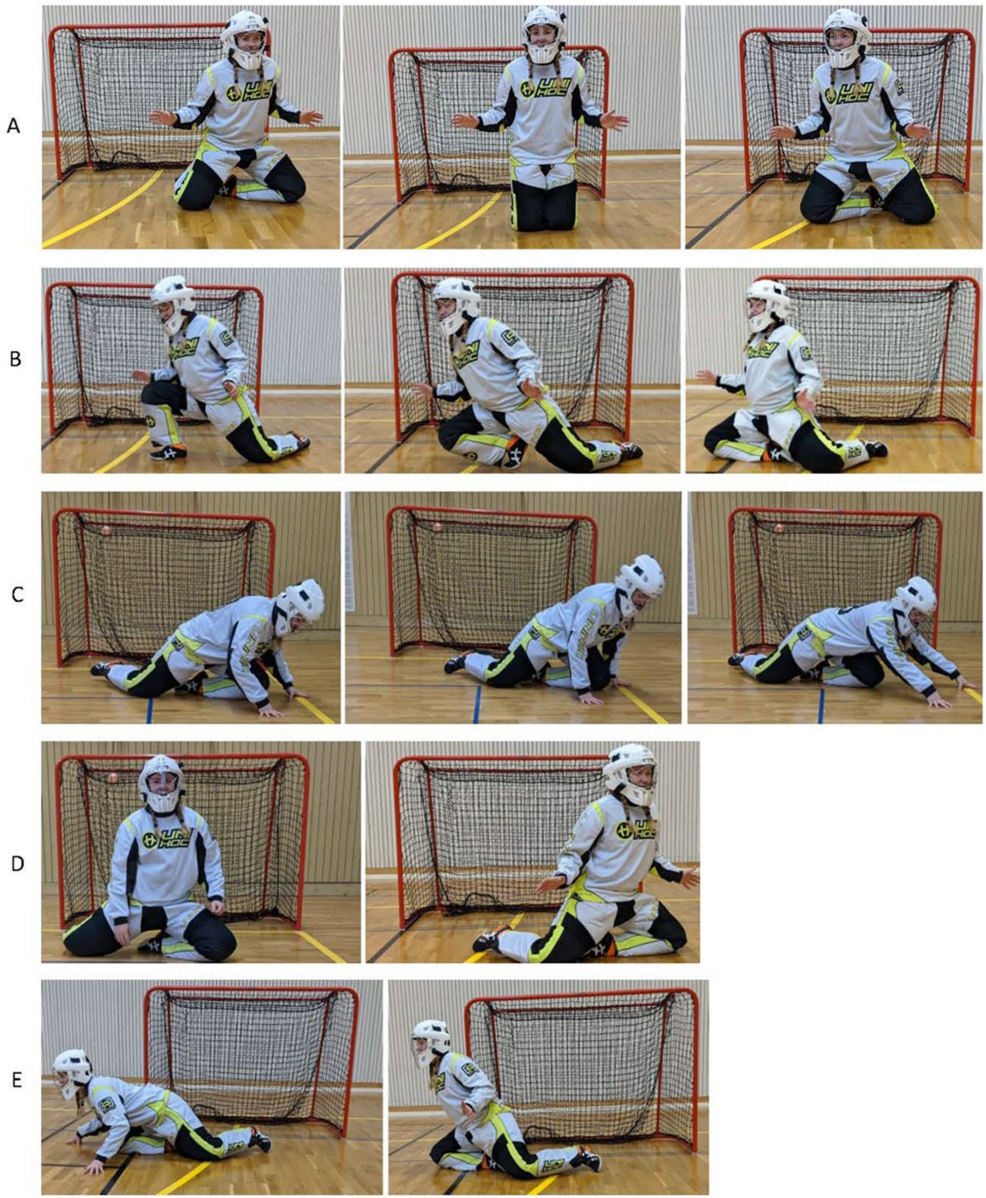
**A-E** illustrates different movements. **a**) ‘Short side movements’ while kneeling occurs when the goalkeeper makes small moves/slide movements to the sides. **b**). ‘Pull’ is when the goalkeeper performs a pull to the right. **c**) ‘Slide while kneeling’ occurs when the goalkeeper performs a slide to one side. **d**) ‘Small leg rotations’ occur when the goalkeeper performs a small rotation of the leg. **e**) ‘Longer ball cover at the goalpost’ is when the goalkeeper performs a longer ball cover in two different ways (low and high)

The authors developed an assessment protocol of different positions and movements that occur during a game through a primary collective video analysis. After this, the protocol was discussed with floorball practitioners. Game movements were defined and explained (see Table 1; Fig. 1). The duration of different positions—on the knees, quadruped position, and standing—were reported in minutes. Different movements were counted; i.e., the number of times the goalkeepers performed a specific movement. In addition, other movements were noted in the protocol to capture individual differences.

**Table 1:** Description of positions and movements observed during gameplay

Positions	Movements
• *Kneeling*—the goalkeeper standing with both knees or standing with both knees and with one hand on the floor or standing with one knee and one foot on the floor. • *Quadruped position*—the goalkeeper is standing with both knees and hands in contact with the floor. • *Standing*—standing on both feet.	• *Short side movements while kneeling*—the goalkeeper makes small moves with the knees or small slides to the side in one direction (see Fig. 1A). In the same movement direction, multiple small steps/slides can occur, but this was counted as a single sequence. • *Pull*—the goalkeeper moves by lifting up one leg and pulling in the same direction as the other leg. For example, the goalkeeper lifts her right leg and pull the body to the right (see Fig. 1B). • *Standing up*—moving from a kneeling to a standing position. • *Slide*—the goalkeeper pushes herself backwards with her hands (see Fig. 1C). • *Forward movement*—the goalkeeper slides forward in the game path direction, either in a short side movement, or pull, push, or slide. • *Push*—the goalkeeper moves by lifting one leg and pushing herself in the other direction; for example, lifting the right leg and pushing to the left. • *Small leg rotations*—the goalkeeper makes a rotation with the hip and lower legs (see Fig. 1D). • *Longer ball cover at the goalpost*—during the game the goalkeeper stands at the front goalpost in the corner and places one leg toward the back goalpost to cover the goal line (see Fig. 1E). Could be formed in two different ways (low and high). • *Dive*—in order to make a save, the goalkeeper makes a dive, landing on the side of the body, or on the trunk.

The videos were analysed by two authors separately. To increase reliability, the films were analysed several times by the same person but on different occasions, and the two authors observed each other´s videos to see whether the results were consistent. Disagreements were discussed and resolved with a joint decision. The game consists of three periods, and the effective playing time was 20 min per period. Total game times varied, which lead to different total playing times for each goalkeeper. Longer breaks in the game occurred due to timeout, injuries, goals, and/or penalties. The different movements and positions that occurred during these breaks were not included in the observation.

### Statistical analysis

Excel and Statistical Package for the Social Sciences (IBM SPSS Statistics, Armonk, New York, USA), version 28 was used for the statistical analyses. Total game time for the goalkeepers was reported in minutes. Since game times varied, the time spent kneeling, in the quadruped position, and the time spent standing were presented in percent of total game time. Nine different movements were analysed. The number of performed movements and mean (SD) were reported. For the five most common movements, the individual number of movements were reported. Due to variation in gameplay (offensive versus defensive), total playing time and the time spent in kneeling position differed between goalkeepers, therefore the number of movements per minute of total kneeling time was reported.

## Results

Playing time were in average 67 (SD15) minutes. Two of the twelve goalkeepers played 28 and 43 min, respectively, and the remaining ten goalkeepers played between 70 and 76 min.

### Positions

**Fig. 2:**
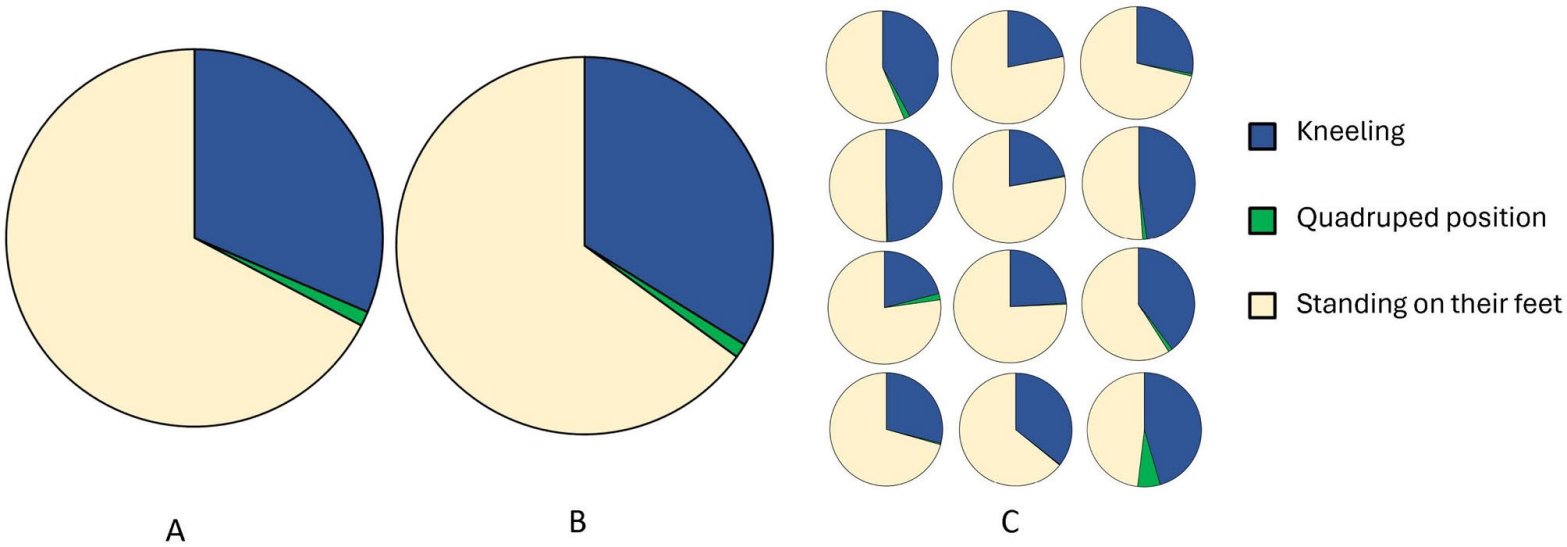
**A-C** **a** Distribution of playing time between kneeling, quadruped position, and standing for goalkeepers who played more than 70 min. **b** Distribution of playing time between kneeling, quadruped position and standing on their feet for all 12 goalkeepers. **c** Individual distribution of the three different positions for all 12 goalkeepers

Three different positions were observed during the game, namely, kneeling, quadruped position, and standing on their feet. The ten goalkeepers that played more than 70 min were on their knees 31.4% (SD 10.1%) of playing time, in the quadruped position 1.3% (SD 1.9%) of the time, and were standing 67.3% (SD11.2%) of playing time (Fig. 2A). Figure 2B are presenting the distribution for all twelve goalkeepers. Kneeling time varied between the twelve individuals (Fig. 2C) where the goalkeeper with highest percentage spent 49% of the total playing time on their knees. The goalkeeper with the lowest amount of kneeling time spent 21% of the total playing time on their knees.

### Movements

The 12 goalkeepers performed a total of 6237 movements of the 9 movements included in the protocol. Five of the nine observed movements were performed more frequently. These five movements were short side movements, pull, stand up, small rotation with legs, and longer ball covers at the goalpost (Table 2).

**Table 2:** Number and mean (SD) of the nine movements included in the protocol

Movements	Number of events	Mean (SD)
Short side movements	2031	169 (69)
Pull	1084	90 (40)
Standing up	993	83 (24)
Small leg rotations	647	54 (29)
Longer ball cover at the goalpos*t*	616	51 (21)
Slide	276	23 (25)
Movements forwards	273	23 (15)
Push	226	19 (9)
Dive	91	8 (5)

A great variation in movement was seen regarding how many and what type of movements each goalkeeper performed during the game (Table 3). The goalkeeper with the highest number of movements per minute of total kneeling time performed 34 movements per minute, while the goalkeeper with the lowest number performed 14 movements per minute.

**Table 3:** The five most common movements during the games reported in mean (SD). Different goalkeepers’ individual total number of movements and movements per minute of time kneeling. Due to variation in gameplay (offensive versus defensive), total playing time and time spent kneeling differed between goalkeepers, number of movements per minute of total kneeling time were reported

Movement	1	2	3	4	5	6	7	8	9	10	11	12	Mean (SD)
Kneeling time (min)	18.1	14.0	15.0	21.1	16.0	16.8	18.3	26.7	19.7	33.4	29.6	34.0	21.9 (7.2)
Total movements Number Movements/min	425 23	201 14	319 2	445 21	535 34	398 24	356 19	496 19	487 25	543 16	626 21	540 16	448 (117) 21 (5)
Short side movements Number Movements/min	196 11	95 7	121 8	157 7	187 12	124 7	80 4	216 8	101 5	200 6	316 11	238 7	169 (69) 8 (2)
Pull Number Movements/min	68 4	31 2	30 2	97 5	104 7	81 5	77 4	87 3	158 8	154 5	113 4	84 2	90 (40) 4 (2)
Standing up Number Movements/min	59 3	31 2	68 5	94 4	109 7	110 7	104 6	75 3	80 4	90 3	103 3	70 2	83 (24) 4 (2)
Small leg rotations Number Movements/min	57 3	14 1	42 3	30 1	43 3	65 4	45 2	45 2	125 6	50 1	41 1	90 3	54 (29) 3 (1)
Longer ball cover at the goalpost Number Movements/min	45 2	30 2	58 4	67 3	92 6	18 1	50 3	73 3	23 1	49 1	53 2	58 2	51 (21) 3 (1)

The movement with greatest individual variation was short side movements (Table 3), where goalkeeper number eleven performed 316 in comparison to goalkeeper number seven, who only performed 80 movement. When taking kneeling time into account, goalkeeper number eleven performed 11 short side movements per kneeling minute, in comparison to goalkeeper number seven, who performed 4 short side movements per minutes. Another movement with a large individual variation was pull (Table 3), where goalkeeper number nine made 8 pulls per minute, compared to goalkeeper number three, who made two pulls per minutes.

## Discussion

There is a lack of knowledge about floorball goalkeepers and their gameplay movements. Therefore, the aim of this study was to observe the type and frequency of female floorball goalkeepers’ movements during a game. Our results showed that female floorball goalkeepers spend a significant amount of their playing time in a kneeling position. The goalkeeper with the highest number of movements per minute performed 34, while the goalkeeper with the lowest number of performed 14 per minute of total kneeling time. During the observation, several individual variations were seen. Some of the variation depended on the match situation; namely, whether the team was offensive or defensive. More movements and more time spent in the kneeling position were observed when playing against an offensive team. Some goalkeepers were more forceful, while others were softer in their movement combinations.

The present study provides an overview of the game movements of floorball goalkeepers, indicating that the five following movements—short lateral movements, pulls, lifts, small leg rotations, and longer ball covers at the post—were the most common and performed the most often. It is difficult to compare floorball to other sports due the different characteristics of the gameplay, however, there are some similarities to the movements of ice hockey goalkeepers. Bell et al. (2008) found that NHL goaltenders most frequently move vertically, laterally, and out of the net to play the puck during a game [15]. Floorball goalkeepers spend more time kneeling and make more short side movements. The time spent kneeling distinguishes floorball goalkeepers’ movements from goalkeepers in other team sports. However, floorball and ice hockey goalkeepers` movements may partially be compared to each other, even though ice hockey is played on a different playing surface with different gear. In both games, many movements are made while kneeling [15, 16], and the goalkeepers use a similar technique, the so-called “butterfly” technique. In ice hockey, the butterfly technique means that the goalkeeper drops to their knees and internally rotates their hips so that the lower extremity’s padding is parallel to the ice [16]. This technique requires extensive hip flexion and internal rotation. In floorball, the “butterfly” movement is when floorball goalkeepers’ drop to quadruped position and make small rotations with their legs. In a study by Wörner at all (2021), both ice hockey goalkeepers and goalkeeper coaches perceived the butterfly technique as one of the most demanding techniques [11]. These techniques, which forces the joints and muscles of the hip and groin into extreme ranges of motions with repetitive loads, are presumed to increase the risk of hip and groin problems in ice hockey goalkeepers [12].

In addition to observed extreme positions in the hip and groin region, it can be assumed that extensive strain is also put on the knee joints. The analysis showed that short lateral movements could be performed in two ways. Either the goalkeepers took small steps on their knees, which can lead to impacts on the knee joint with each landing, or they performed a sideways slide where the floorball goalkeeper pushed one leg to the right, for example, and the other leg followed. The sliding movement can be gentler when it comes to impact, but, on the other hand, it increases friction. Since these movements are unique to floorball and no similar sport-specific data exist, movements can be partially compared to workers who spend a significant amount of their work tasks in a kneeling position. A review by Schram et al. has shown that these groups have a greater risk of developing knee and hip osteoarthritis [17]. For example, floor and carpet layers who spend a considerable amount of their workday kneeling are at risk of suffering various knee issues due the significant strain placed on their knees. Common injuries reported by carpet layers include bursitis, patellar syndrome, and meniscal tea [18] Canetti et al. confirms that working on one’s knees increases the risk of knee problems [19]. Although floorball goalkeepers do not kneel to the same extent during training and matches, similarities can still be observed. Most goalkeepers choose to use knee pads that provide a softer surface to kneel upon, which can partially lessen the load and friction placed on the knee joints. More knowledge and research on biomechanical factors and protective equipment are needed.

To gain a better understanding of the movements of goalkeepers, we chose to conduct a quantitative observational study. The study design has both methodological strengths and shortcomings. Data collection was done through video recordings to facilitate multiple analyses of the goalkeepers’ movements and to observe the movements in detail. The main limitation was that sometimes it was challenging to be consistent in interpreting the movements, as the goalkeepers occasionally performed combinations of different movements. During video recording, the camera was positioned diagonally away from the stands. When observing the videos, movements when the goalkeeper was facing away from the camera and when many players were in front of the goal were more challenging to analyse. To facilitate observation of the goalkeeper’s movements, the camera could have been positioned directly in front or behind of the goal, mounted on the ceiling. A large sample would have strengthened the result and generalizability.

The result of the study was in line with our hypothesises i.e. that the movements made when defending the goal are done on the knees, and with large amount of short lateral movements, and with a large individual variation among the goalkeepers. Future research should review goalkeepers’ loads during training versus during matches, over a week, and during different seasons of the year (pre-season, match season). In addition, further studies examining individual playing profiles for both female and male floorball goalkeepers are warranted. These studies should also observe transitions between movements and take match situation, offensive and defensive, into account. Continued research investigating the types of injury sustained by goalkeepers and injury incidence, separate from field players, are needed. By identifying the most demanding gameplay movements or those that may be related to specific injuries, targeted preventive measures and training can be developed to reduce the risk of injury. Coaches, goalkeepers, and physiotherapists can use knowledge from the present study when planning and adapting training to more favourable and varied approaches.

## Conclusion

As hypothesised, during a floorball match, goalkeepers spend significant parts of the game on their knees and perform a great number of movements per minute. At the group level, the most common movements were short lateral movements, pulls, lifts, small leg rotations, and longer ball covers at the post. A large variation was seen regarding how many and what type of movements each goalkeeper performed during the game. Further research on how these gameplay aspects may impact load as well as physical needs are warranted. To best of our knowledge this is a first study regarding floorball goalkeepers’ movements, and it will enhance the understanding of floorball goalkeepers’ movements, providing a foundation for future projects focused on injury prevention and specific training programmes.

## Data Availability

The data (videos) analysed during the current study are not publicly available due anonymity of the participants but are available from the corresponding author on reasonable request.
